# Vibration of a Liquid Crystal Elastomer Spring Oscillator under Periodic Electrothermal Drive

**DOI:** 10.3390/polym15132822

**Published:** 2023-06-26

**Authors:** Kai Li, Jiangfeng Lou, Shaofei Hu, Yuntong Dai, Fei Wang, Yong Yu

**Affiliations:** 1 College of Civil Engineering, Anhui Jianzhu University, Hefei 230601, China; kli@ahjzu.edu.cn (K.L.); 13275735791@163.com (J.L.); 19105650998@163.com (S.H.); daiytmechanics@ahjzu.edu.cn (Y.D.); 2 Institute of Energy, Hefei Comprehensive National Science Center, Hefei 230039, China; kingfly@mail.ustc.edu.cn; 3 State Key Laboratory of Mining Response and Disaster Prevention and Control in Deep Coal Mines, Anhui University of Science and Technology, Huainan 232002, China

**Keywords:** liquid crystal elastomer, spring oscillator, vibration, periodic electric field, dynamic LCE model

## Abstract

The oscillations of electrically actuated thermally-responsive liquid crystal elastomer (LCE) microfibers under cyclic electric actuation have been discovered in recent experiments. Periodic electric actuation is a common method of active control with potential applications in the fields of micro-actuators. In this paper, the vibration behavior of LCE spring oscillator under periodic electrothermal drive is studied theoretically. Based on the dynamic LCE model, the dynamic governing equation of the LCE spring oscillator is established, and the time history curves of the vibration are obtained by numerical calculations. The results show that the periodic electrothermal drive can cause periodic vibration of the LCE spring oscillator. With the increase of time rate, the vibration amplitude increases first and then decreases. In a small damping system, there exist optimal sets of electrothermal drive period and electrothermal drive time rate to maximize the system amplitude. For the optimum periodic mode, the vibration amplitude of the spring oscillator is affected by the current heat, damping coefficient, gravital acceleration, spring constant and shrinkage coefficient, but not by the initial velocity. The application examples of LCE materials show that periodic electrothermally driven LCEs have promising applications. The results of this study are instructive for the design of soft robots and LCE-based electric locomotives.

## 1. Introduction

Liquid crystal elastomers (LCEs) are a class of stimuli-responsive polymers that can undergo shape-change in response to external stimuli [1], such as heat [2], electric field [3,4], light [5,6] magnetic field [7] and chemical substances [8]. The shape change of LCEs can generally be programmed during fabricating by orienting the liquid crystal phase prior to crosslinking. Generally, the response of LCE materials to these stimuli is rapid and reversible, and therefore LCE materials have a wide range of promising applications in many fields, such as artificial muscles [9], micro-systems and micro-electromechanical systems [10], actuators and sensors [11], energy harvesters [12], scalable optical devices [13], soft robots [14], new materials [15], etc.

**Figure 1:**
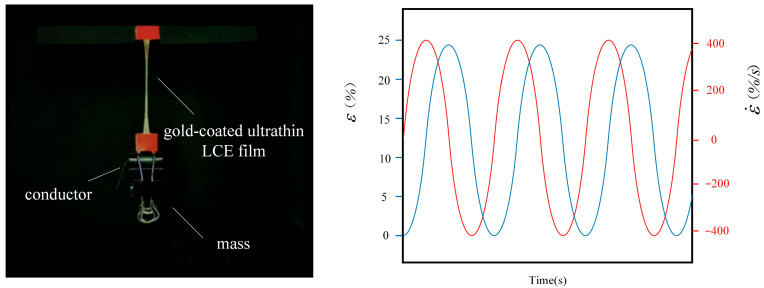
LCE material has periodic motion under periodic electrothermal drive [42].

Vibration of beams, plates and shells induced by excitation of magnetic field, light and heat is widely used in aerospace, mechanical and civil engineering and other fields [16,17,18,19,20,21]. In terms of electric drive, electrothermally driven vibration of LCE materials has attracted the attention of many scholars. It can convert electrical energy into mechanical energy, which can be used for precise control of LCE composite materials [22,23,24] under electro-thermal drive [25]. It can be applied to micro robots [26,27], precision mechanical devices [28,29,30], self-excited mechanics [31], and direct drive without motor [32]. The electrothermally driven vibration [33,34] is caused by thermal sensing deformation of LCE material [35,36,37] due to heat generation by current resistance. For example, a thermodynamic model of rod rolling on a hot surface is built to change the thermal deformation and stress field in the stable rolling process of the rod, so as to further predict the rolling speed [38]. Wang, M. et al. studied LCE electric locomotive to implement an electrically driven cylindrical power system composed of soft brake and conductive track [39,40,41]. As shown in Figure 1, Cai’s group recently achieved through extensive experiments, that LCE materials can undergo periodic motion through phase transition that produce reversible shrinkage [42].

Although many of the above experimental and theoretical studies have studied the deformation and self-excited vibration of LCE materials induced by electric drive [43,44,45,46,47,48,49]. Electrothermal LCE material is recently fabricated and can deform and move dynamically under periodic electrothermal drive [42], which has important application in mechanical energy conversion system, nano-polymer materials [50,51,52,53,54,55], and fiberboard shell [56,57,58,59,60]. Based on the LCE model, a LCE spring oscillator under periodic electrothermal drive is theoretically studied in this paper. Our goal is to achieve the simple mechanical movement by controlling the periodic energization, due to which the heat generated by the resistance causes the apparent thermal deformation of the LCE material to exhibit periodic deformation motion. The proposed LCE spring oscillator has the unique advantages of simple structure, convenient control and high precision, which is expected to have potential application prospects in soft robots [61,62,63], micromachinery [64,65], precision instruments [66,67], polymer chemistry [68,69,70,71,72], tunable intensity modulators [73], dielectric elastomers [74], photonic MEMS actuators [75] and other fields.

The rest of this article is structured as follows. In Section 2, we first propose the dynamic model of a LCE spring oscillator under periodic electrothermal drive, and establish and solve the governing equation. Section 3 discusses the periodic vibration under optimal conditions in detail. In Section 4, the effects of system parameters on the vibration amplitude of the spring oscillator are discussed in detail. Finally, we conclude in Section 5.

## 2. Model and Theoretical Formulation

In this section, the dynamic governing equation of the spring oscillator is derived by constructing the LCE material model, and the formulas in cases of power-on and power-off are derived respectively, which are then transformed into dimensionless formulas and solved by the governing equation.

### 2.1. Dynamic Governing Equation of Spring Oscillator Stimulated by Electrothermal Drive

**Figure 2:**
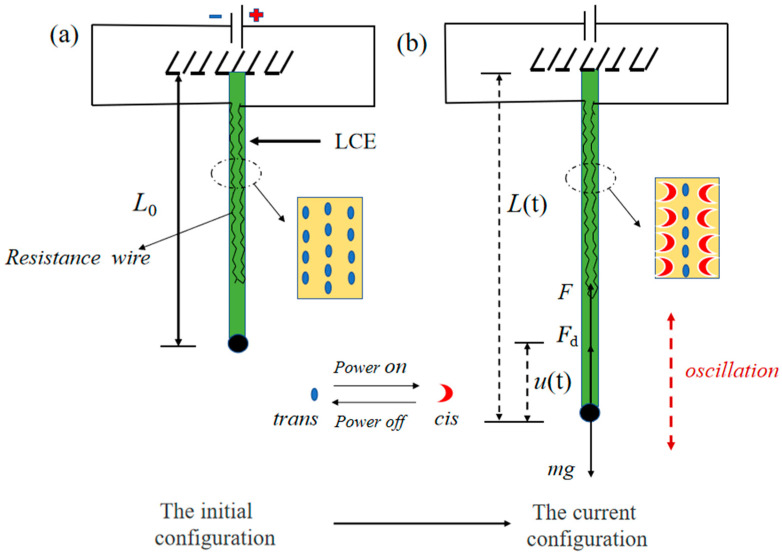
Schematic diagram of a LCE spring oscillator under periodic electrothermal drive. (**a**) The initial configuration; (**b**) The current configuration.

As shown in Figure 2a, a LCE fiber with original length $L_{0}$ is fixed at the fixed end, and a resistance wire is placed in the LCE fiber. Following the works of fabricating LCE made of LC monomer (RM257) and cross-linker (PETMP), etc., by the two-step cross-linking reaction reported by Yakacki et al. [76], one can first fabricate a loosely cross-linked LCE fiber in a tube with an inner curved resistance wire, and then fully cross-link the LCE fiber after being taken out of the tube and tensioned. Then, a ball with mass *m* is attached to the free end of the LCE fiber. The displacement coordinate system is established by taking the place where the fiber connects the ball as the coordinate origin. The initial velocity of the ball is *v*. During the vibration process, the instantaneous length of the LCE fiber can be denoted as L(t), and the instantaneous displacement of the ball can be represented by u(t), as shown in Figure 2b.

When the resistance wire is energized, the temperature rises causing the LCE fiber to shrink and deform. When the power is removed, the electrothermally driven shrinkage of LCE fiber will recover. When a periodic electrothermal drive is applied, the temperature will rise and fall periodically to make the ball vibrate up and down periodically. The masses of the LCE fiber and resistance wire are much smaller than the mass of the ball, so they are ignored in the calculations. As presented in Figure 2b, the mechanical analysis of the ball shows that the ball is subjected to the tension of the LCE fiber F(t), the gravity of the ball itself and the damping force $F_{d}=CV$, where C represents the damping coefficient and *V* is the velocity of ball. For the convenience of later calculations, it is assumed that the damping is proportional to the velocity of the ball, and in the direction opposite to the motion direction of the ball. According to Newton’s second law, the mechanical governing equation for the ball can be obtained as:

(1) $$m\frac{d^{2}u(t)}{dt^{2}}=mg-F\left(t\right)-C\frac{du\left(t\right)}{dt},$$

where g represents the gravital acceleration.

In this paper, the linear elastic model is adopted, where the spring force of LCE fiber is proportional to the length variation. In Equation (1), the tension of LCE fiber can be expressed as:

(2) $$F\left(t\right)=K\left[u\left(t\right)-L_{0}\varepsilon \left(t\right)\right],$$

where K is the spring constant, and ε(t) refers to the electrothermally driven shrinkage strain of LCE with an assumption that it is linearly related to the temperature change *T*(*t*) in the LCE fiber and can be written as:

(3) ε(t)=−αT(t),

where α is the shrinkage coefficient.

### 2.2. Electrothermally Driven Temperature Field in LCE Fiber

The radius of LCE fiber is very small, which is known as very small ratio. Therefore, it is assumed that the heat exchange within the electrically stimulated LCE spring oscillator is very fast, and the temperature within the spring oscillator is uniform. According to Joule’s law, current can be converted into heat during conduction. Suppose that the system composed of a resistance wire and a LCE is a pure resistive circuit system, the electrothermal is the energy transferred due to the temperature change *T*, and always from the hot body to the cold body. A new temperature equilibrium has been reached, with the equation being establish as:

(4) $$\frac{dT}{dt}=\frac{q-kT}{\rho_{C}},$$

where $\rho_{c}$ is the specific heat capacity, q represents the electricity heat, and k represents the heat transfer coefficient. Equation (4) can be rewritten as

(5) $$\frac{dT}{dt}=\frac{1}{\tau }\left(\frac{q}{k}-T\right),$$

where $\tau =\frac{\rho_{C}}{k}$ is the thermal relaxation time.

By solving Equation (5), we have:

(6) $$T=\frac{q}{k}(1-e^{-t/\tau }).$$

When no electricity is applied, the temperature in LCE will gradually decrease. When the current is removed, it can be obtained:

(7) $$T=\frac{q}{k}e^{-t/\tau }.$$

Combining Equations (4)–(7), and defining the dimensionless temperature change $\bar{T}=T/T_{e}$(*T*_e_ is the environmental temperature), the dimensionless electricity heat $\bar{q}=q/kT_{e}$ and the dimensionless time $\bar{t}=t/\tau$, the dimensionless forms of temperature change in electrically stimulated LCE are derived for the following two cases.

Case Ι: the electrically stimulated LCE is energized, named as power-on state:

(8) $$\overset{\_\_}{T}=\bar{q}\left(1-e^{-\bar{t}}\right).$$

Case II: electrically stimulated LCE is out of electricity, named as the power-off state:

(9) $$\overset{\_\_}{T}=\bar{q}e^{-\bar{t}}.$$

### 2.3. Nondimensionization

For ease of calculation, the following dimensionless parameters are defined as $\bar{F}\left(t\right)=F\left(t\right)\tau^{2}/mL_{0}$, $\bar{u}(t)=u(t)/L_{0}$, $\bar{C}=C\tau /m$, $\bar{g}=g\tau^{2}/L_{0}$, $\bar{v}=vL_{0}/\tau$, and $\bar{K}=K\tau^{2}/m$. Therefore, Equation (1) is simplified to the dimensionless form:

(10) $$\frac{d^{2}\bar{u}\left(\bar{t}\right)}{d{\bar{t}}^{2}}=\bar{g}-\bar{K}\left[\bar{u}(\bar{t})+\alpha \bar{T}\right]-\bar{C}\frac{d\bar{u}\left(\bar{t}\right)}{d\bar{t}}.$$

Combining Equations (6)–(10), we can obtain:

Case Ι: power-on state:

(11) $$\frac{d^{2}\bar{u}\left(\bar{t}\right)}{d{\bar{t}}^{2}}=\bar{g}-\bar{K}\left\{\bar{u}(\bar{t})+\alpha \bar{q}(1-e^{-\bar{t}})\right\}-\bar{C}\frac{d\bar{u}\left(\bar{t}\right)}{d\bar{t}}.$$

Case II: power-off state:

(12) $$\frac{d^{2}\bar{u}\left(\bar{t}\right)}{d{\bar{t}}^{2}}=\bar{g}-\bar{K}\left\{\bar{u}(\bar{t})+\alpha \bar{q}e^{-\bar{t}}\right\}-\bar{C}\frac{d\bar{u}\left(\bar{t}\right)}{d\bar{t}}.$$

We can obtain the dynamic response of the LCE spring oscillator by iterative method, that is the displacement and velocity of the ball versus time. Equation (10) controls the periodic motion of the spring oscillator under periodic electrothermal drive. In order to solve these complex differential equations with variable coefficients, the Runge-kutta method is used and numerical calculations are carried out in matlab software. Equations (11) and (12) control the dynamics of LCE spring oscillator in the power-on and power-off states, respectively. The temperature change in LCE under power-on and power-off states are described by Equations (8) and (9), respectively.

## 3. Results and Discussion

Based on the governing Equations (10)–(12), the force vibration of the spring oscillator under periodic electrothermal drive can be numerically calculated. To diminish the deviations of experimental results from the ideal theoretical situation, the resistance wire in the experimentation should be thin enough to reduce the constraint on the LCE fiber. Meanwhile, LCE fiber should be slender enough to satisfy the one-dimensional hypothesis of the model. In addition, a circuit controller is required to accurately and periodically turn on and turn off the circuit. In a steady ambient, the room temperature should also be low enough for quick heat exchange. In this section, by calculating the forced vibration of the spring oscillator under periodic electrothermal drive, the optimal electrothermal drive period and the optimal electrothermal drive time rate for the spring oscillator under electric stimulation are discussed. Then the following parameters for achieving the optimum periodic mode are discussed, as well as the temperature change versus time, force change versus time, force versus displacement and the work change of damping with displacement.

### 3.1. Forced Vibration of LCE Spring Oscillator under Periodic Electrothermal Drive

In this section, forced vibration of the LCE spring oscillator under periodic electrothermal drive will be discussed. Periodic eletrothermal drive is a relatively simple and common mode of active control, and it is therefore particularly important to master its control law. In this section, the optimal period and the optimal time rate are determined by numerical calculations based on the criterion of maximizing the steady-state vibration amplitude of the spring oscillator. After the optimum periodic mode is achieved, the effects of heat energy, damping coefficient, gravital acceleration, spring constant, shrinkage coefficient and initial velocity on the amplitude are studied respectively.

In order to study the periodic motion of the LCE spring oscillator under periodic electrothermal drive, the typical values of the dimensionless parameters need to be estimated accordingly. Based on the available experiments [42,77], typical material properties and geometric parameters are listed in Table 1, and the corresponding dimensionless parameters are listed in Table 2.

**Figure 3:**
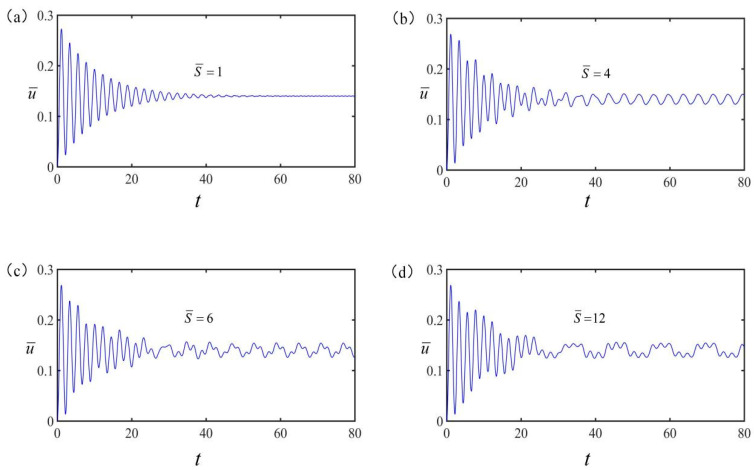
Forced vibration of the ball at the free end of LCE spring oscillator under different electrothermal driving periods. (**a**) Forced vibration under $\bar{S}=1$; (**b**) Forced vibration under $\bar{S}=4$; (**c**) Forced vibration under $\bar{S}=6$; (**d**) Forced vibration under $\bar{S}=12$. The amplitude of spring oscillator is different under different electrothermal driving periods.

Figure 3 shows the forced vibration of the LCE spring oscillator under different dimensionless electrothermal driving periods $\bar{S}$, which is defined as $\bar{S}=S/\tau$, with S is the electrothermal driving period. The other parameters are set as $\bar{v}=0$, $\bar{C}=0.25$, $\bar{q}=0.5$, $\bar{g}=1.2$, $\bar{K}=8$, α=0.2 and electrothermal drive time rate ${\bar{S}}_{1}/\bar{S}=0.5$, where ${\bar{S}}_{1}$ is dimensionless non-electrothermal driving time in a period and is defined as ${\bar{S}}_{1}=S_{1}/\tau$ with $S_{1}$ is the non-electrothermal driving time in a period. As shown in Figure 3, the vibration of the spring oscillator is periodic and becomes stable after a period of non-periodic vibration. Obviously, the vibration is initially composed of free vibration and pure forced vibration. Due to the damping, the free vibration will disappear, eventually making the spring oscillator only exhibit pure forced vibration as a steady-state vibration. From the figure, the period of the steady-state vibration is consistent with the electrification period, which has a significant effect on the vibration time. As shown in Figure 3a,b, when the electrification period is within four times the natural period, the vibration presents a single-peak steady-state vibration. As shown in Figure 3c,d, when the electrification period is four times longer than the natural period, the vibration presents a multi-peak steady-state vibration. For the sake of later discussion, we will only discuss the single-peak steady-state vibration. The multi-peak steady-state vibration is relatively complex and will not be discussed in this paper. For the single-peak steady-state vibration, the amplitude and equilibrium position are defined as $\left(u_{\max}-u_{\min}\right)/2$ and $\left(u_{\max}+u_{\min}\right)/2$ for convenience. The $u_{\max}$ and $u_{\min}$ in the expression refer to the maximum and minimum values of the vibration displacement at the tip of the steady-state vibrating spring oscillator.

**Figure 4:**
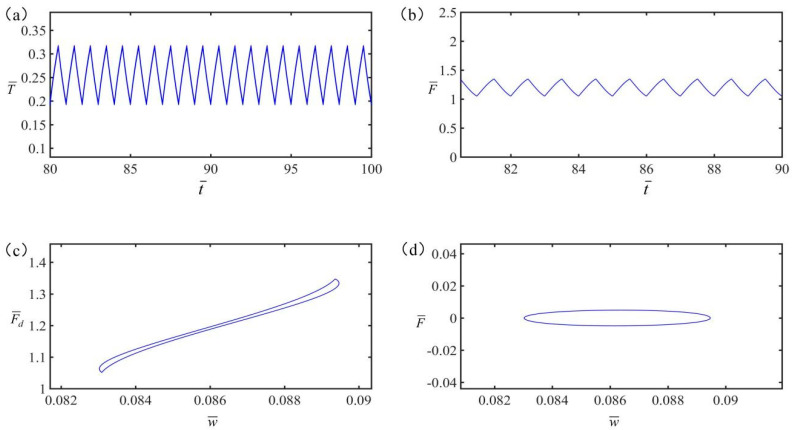
(**a**) Temperature versus time; (**b**) Tension in LCE versus time; (**c**) Damping force versus displacement; (**d**) Tension in LCE versus displacement.

The other parameters in the calculation for Figure 4 are $\bar{v}=0$, $\bar{q}=0.5$, $\bar{C}=0.25$, α=0.2, $\bar{g}=1.2$, and $\bar{K}=8$. Figure 4a,b show that both the temperature and the tension in LCE exhibit a steady periodic variation with time. Figure 4c,d depict the variation of tension and damping force with displacement, representing the work done by the tension and damping force, respectively. Under periodic electrothermal drive, both temperature and tension change periodically with time.

### 3.2. Optimal Electrothermal Drive Period

**Figure 5:**
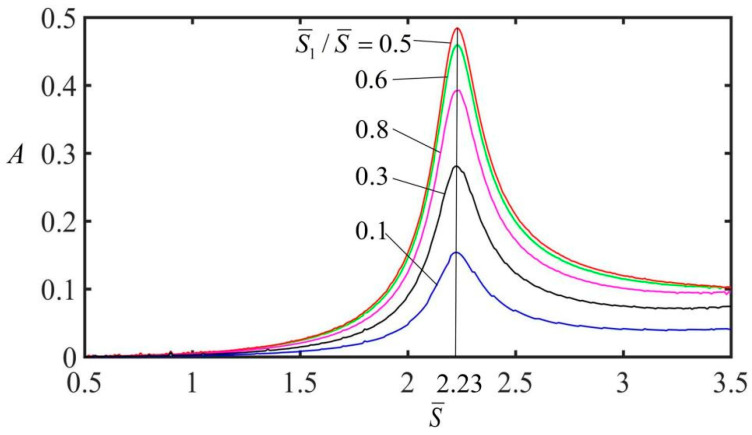
Variation of the amplitude at the free end of the spring oscillator with the electrothermal drive period. With the increasing electrothermal drive period, the vibration amplitude of the spring oscillator first increases and then decreases, with a maximum amplitude when the natural period is 2.23.

As shown in Figure 5, the amplitude at the free end of the spring oscillator varies with the electrothermal drive period when the electrothermal drive time rate ${\bar{S}}_{1}/\bar{S}$ is 0.5, 0.6, 0.3, 0.8 and 0.1 respectively. The other parameters are: $\bar{q}=0.5$, $\bar{C}=0.25$, $\bar{g}=1.2$, α=0.2, $\bar{K}=8$, and $\bar{v}=0$. As the electrothermal drive period increases, the vibration amplitude of the spring oscillator first increases and then decreases, reaching a maximum amplitude, when the natural period is 2.23. It should be noted here that the optimal electrothermal drive period does not change with the electrothermal drive time rate.

### 3.3. Optimal Electrothermal Drive Time Rate

**Figure 6:**
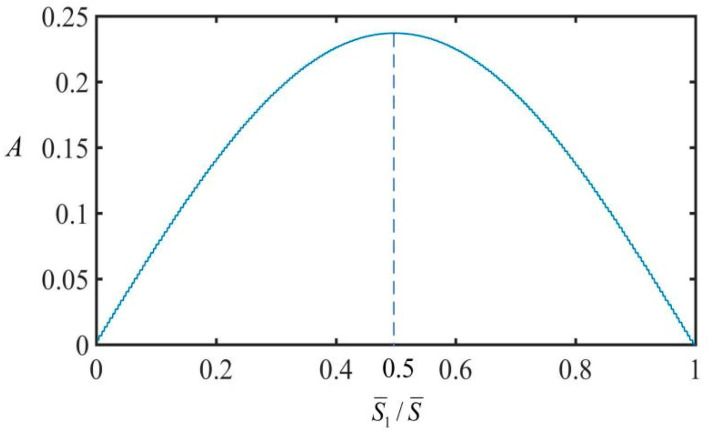
Variation of spring oscillator amplitude with optimal electrothermal drive time rate. The optimal electrothermal drive periodic rate is ${\bar{S}}_{1}/\bar{S}=0.5$.

Figure 6 shows the variation law of vibration amplitude and equilibrium position of the spring oscillator with respect to the optimal electrothermal drive time rate. Other parameters include $\bar{v}=0$, $\bar{q}=0.5$, $\bar{C}=0.25$, α=0.2, $\bar{g}=1.2$, and $\bar{K}=8$. As can be seen from the figure, the amplitude of the spring oscillator is symmetric about the time rate point ${\bar{S}}_{1}/\bar{S}=0.5$, and it reaches its maximum at the symmetric point. With the increase of electrothermal drive time rate, the amplitude of the spring oscillator increases first and then decreases.

.

## 4. Parametric Analysis

During the process of forced vibration, the electrothermal drive heat energy, damping coefficient, spring constant, gravital acceleration, shrinkage coefficient and initial velocity will affect the amplitude and limit cycle of the system. Through parametic analysis, the general laws for the spring oscillator under electric stimulation will be explored in this section.

### 4.1. Effect of the Electricity Heat

**Figure 7:**
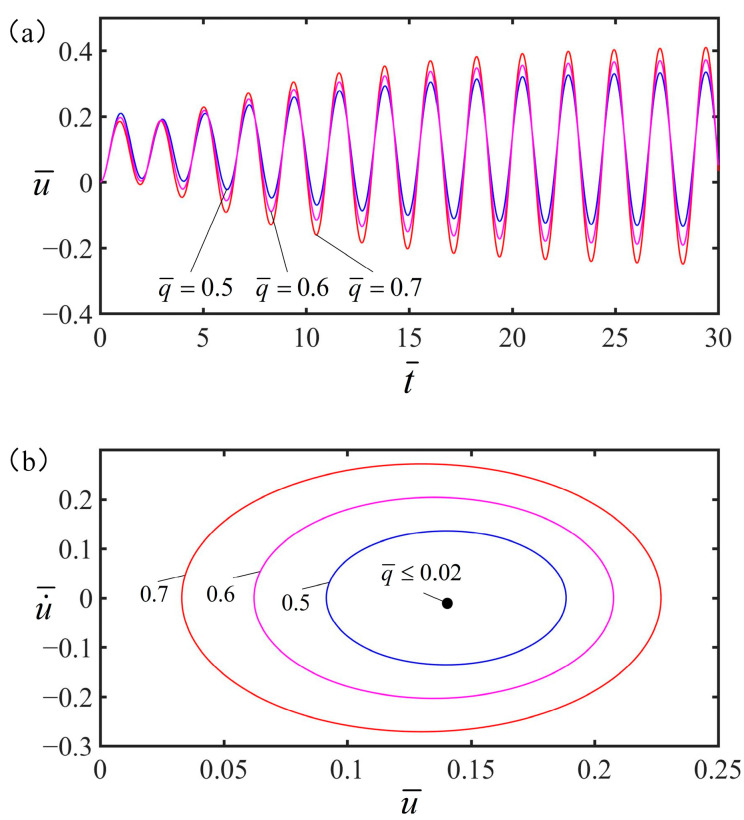
The effect of electric heat on the amplitude of forced vibration. In this case, other parameters are $\bar{v}=0$, $\bar{C}=0.25$, α=0.2, $\bar{g}=1.2$, $\bar{K}=8$, and ${\bar{S}}_{1}/\bar{S}=0.5$. (**a**) The amplitude of forced vibration increases as the heat of the current increases. (**b**) Limit cycle.

Figure 7 shows the relationship between the amplitude at the free end of the spring oscillator and time under different electrothermal drive heat energy $\bar{q}$. The system is in the optimal electrothermal drive period $\bar{S}=2.23$ and the optimal electrothermal drive time rate ${\bar{S}}_{1}/\bar{S}=0.5$, and the other parameters are $\bar{v}=0$, $\bar{C}=0.25$, α=0.2, $\bar{g}=1.2$, $\bar{K}=8$. As observed from Figure 7b, the spring oscillator is at rest when $\bar{q}<0.02$. While $\bar{q}\geq 0.02$, the spring oscillator is in forced vibration. It can be seen that in a periodic electrothermal drive, increasing the heat of the electrothermal drive increases the amplitude, but does not change the vibration time.

### 4.2. Effect of the Damping Coefficient

**Figure 8:**
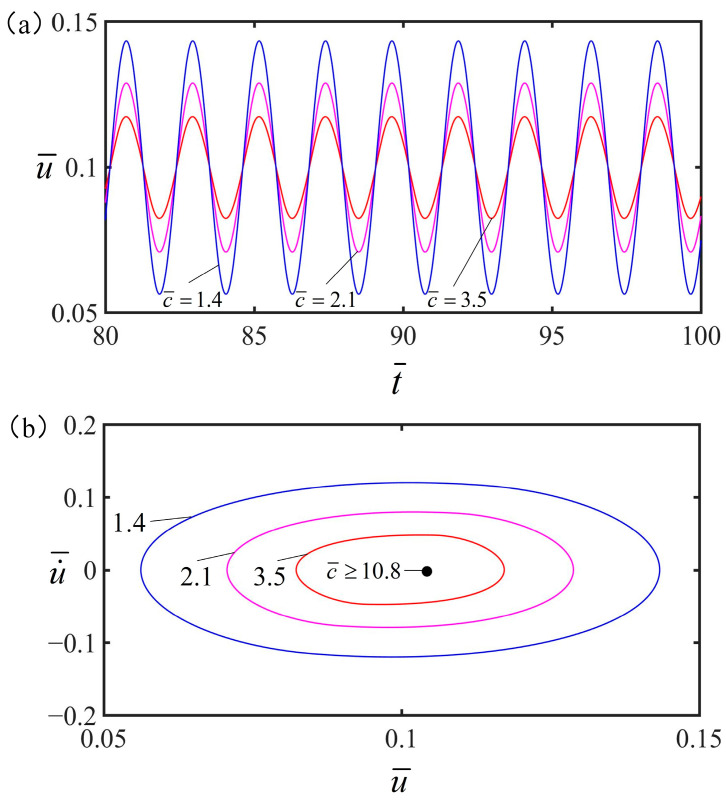
The effect of damping coefficient on the vibration of the spring oscillator under electrothermal drive, In this case, other parameters are $\bar{q}=0.5$, α=0.2, $\bar{g}=1.2$, $\bar{K}=8$, $\bar{v}=0$, and ${\bar{S}}_{1}/\bar{S}=0.5$. (**a**) The amplitude of forced vibration decreases with the increasing damping coefficient. (**b**) Limit cycle.

Figure 8 illustrates the effect of damping coefficient on the vibration of the spring oscillator, with other parameters being $\bar{q}=0.5$, α=0.2, $\bar{g}=1.2$, $\bar{K}=8$, $\bar{v}=0$, and ${\bar{S}}_{1}/\bar{S}=0.5$. Figure 8a presents the decreasing trend of the vibration amplitude with the increasing damping coefficient, which can be explained by the law of energy conservation. The periodic electrothermal drive provides heat energy to compensate for the energy consumed by damping. During the steady-state vibration, the heat energy is the same as the energy dissipated by damping. Energy is conserved throughout the process, and as the damping increases, the more energy it consumes, the less energy is left to ddrive the vibration of the spring oscillator. From Figure 8b, the spring oscillator is at test when $\bar{C}\geq 10.8$ rest. While $\bar{C}<10.8$, the spring oscillator is in forced vibration. It can be seen that under periodic electrothermal drive, damping coefficient plays an important role in periodic electrothermal drive vibration.

### 4.3. Effect of the Gravital Acceleration

**Figure 9:**
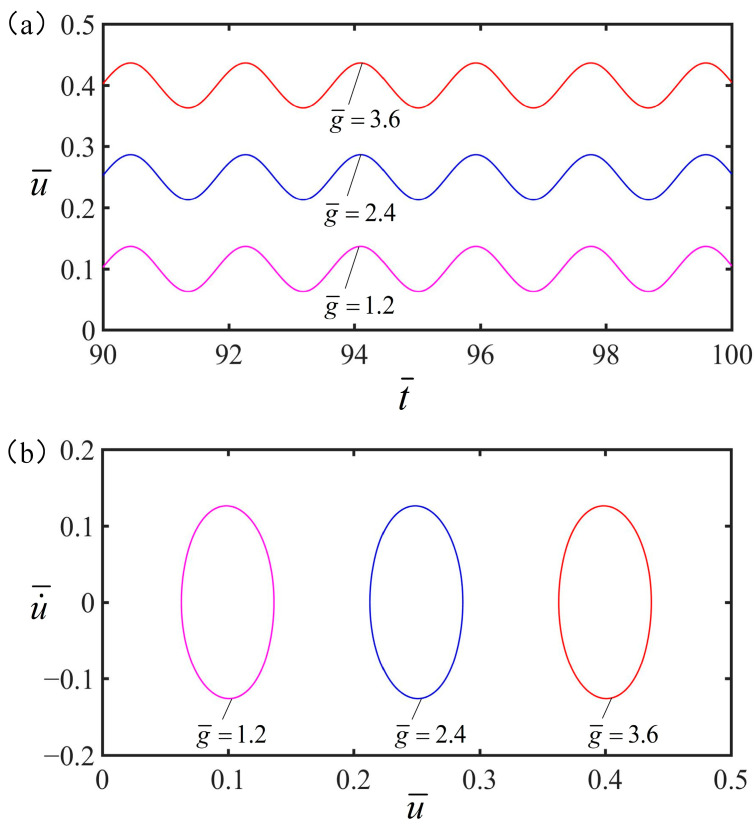
The effect of gravital acceleration on the vibration of the spring oscillator. In this case, other parameters are $\bar{q}=0.5$, $\bar{C}=0.25$, α=0.2, $\bar{K}=8$, $\bar{v}=0$, and ${\bar{S}}_{1}/\bar{S}=0.5$. (**a**) As the gravittional acceleration increases, the equilibrium position of forced vibration gradually increases. (**b**) Limit cycle.

Figure 9 plots how the gravital accelerational influencing the vibration of the spring oscillator under the optimal electrothermal drive time rate. The parameters are set as $\bar{q}=0.5$, $\bar{C}=0.25$, α=0.2, $\bar{K}=8$, $\bar{v}=0$, and ${\bar{S}}_{1}/\bar{S}=0.5$. Figure 9a shows that the equilibrium position of the forced vibration rises as the gravital acceleration increases. As can be seen from Figure 9b, when gravity acceleration exists, the whole spring vibration subsystem will be in a state of forced vibration, gravity acceleration has an important effect on periodic electrothermal drive vibration.

### 4.4. Effect of the Spring Constant

**Figure 10:**
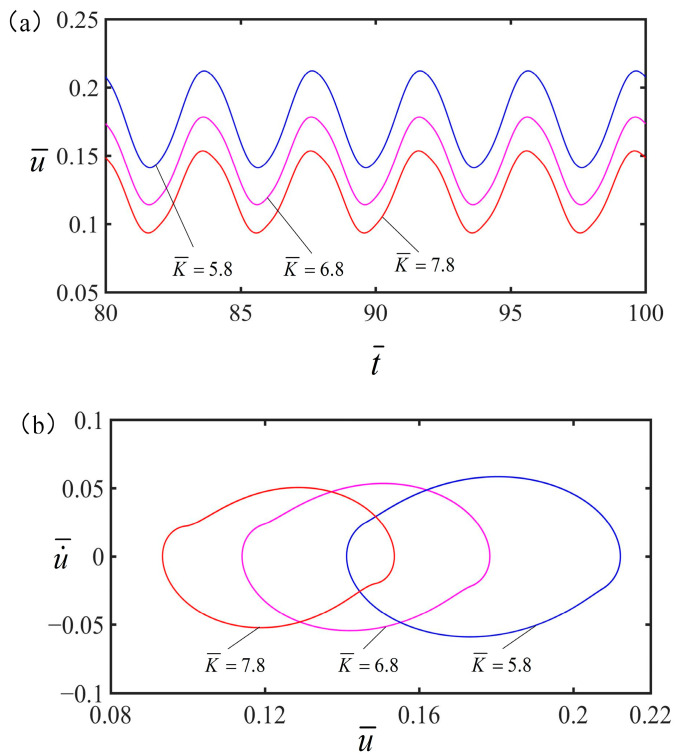
The effect of spring constant on the vibration of the spring vibrator. In this case, other parameters are $\bar{q}=0.5$, $\bar{C}=0.25$, α=0.2, $\bar{g}=1.2$, $\bar{v}=0$, and ${\bar{S}}_{1}/\bar{S}=0.5$. (**a**) The amplitude of the forced vibration decreases as the spring constant increases. (**b**) Limit cycle.

The influence of the spring constant on the vibration of the spring oscillator at the optimal electrothermal drive time rate is displayed in Figure 10, with the parameters being $\bar{q}=0.5$, $\bar{C}=0.25$, α=0.2, $\bar{g}=1.2$, $\bar{v}=0$, and ${\bar{S}}_{1}/\bar{S}=0.5$. From Figure 10a, the amplitude of the forced vibration is observed to decrease as the spring constant increase. As can be seen from Figure 10b, when the spring constant exists, the spring oscillator will be in a state of forced vibration. It can be seen that under periodic electrothermal drive, spring constant plays an important role in periodic electrothermal drive vibration.

### 4.5. Effect of the Shrinkage Coefficient

**Figure 11:**
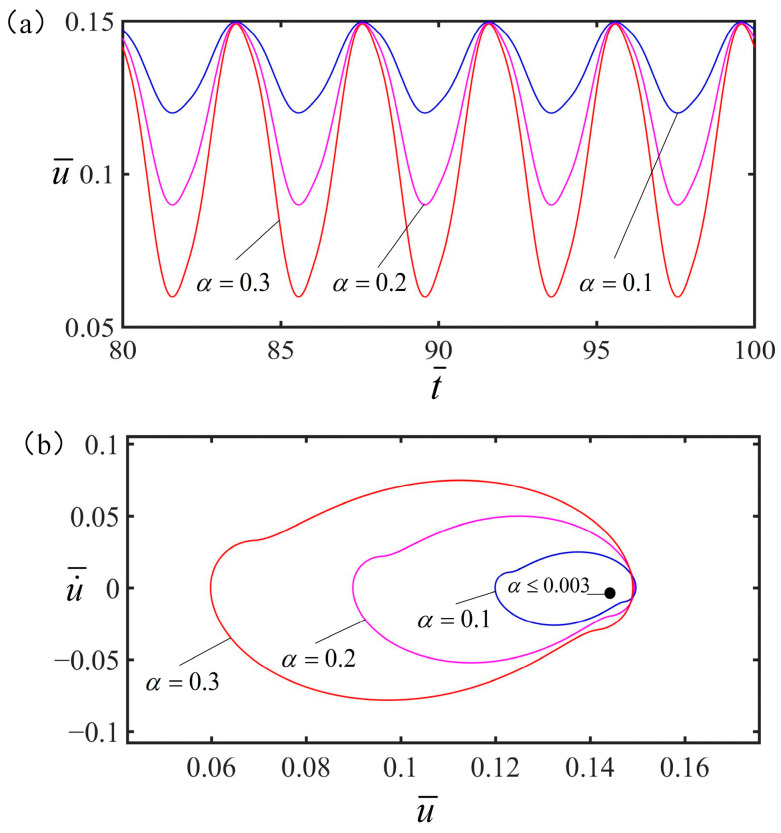
The effect of shrinkage coefficient on the vibration of the spring vibrator. In this case, other parameters are $\bar{v}=0$, $\bar{q}=0.5$, $\bar{C}=0.25$, $\bar{g}=1.2$, $\bar{K}=8$, and ${\bar{S}}_{1}/\bar{S}=0.5$. (**a**) The amplitude of forced vibration increases gradually with the increase of shrinkage coefficient. (**b**) Limit cycle.

Figure 11 plots the influence of the shrinkage coefficient on the vibration of the spring oscillator at the optimal electrothermal drive time rate. The other parameters are set as $\bar{v}=0$, $\bar{q}=0.5$, $\bar{C}=0.25$, $\bar{g}=1.2$, $\bar{K}=8$, and ${\bar{S}}_{1}/\bar{S}=0.5$. It is clear in Figure 11a that the vibration amplitude of the spring oscillator increases with the increase of shrinkage coefficient. As can be seen from Figure 11b, the spring oscillator is at rest when α≤0.003, while in forced vibration when α>0.003. It can be seen that in a periodic electrothermal drive, increasing the shrinkage coefficient of the electrothermal drive increases the amplitude, but does not change the vibration time.

### 4.6. Effect of the Initial Velocity

**Figure 12:**
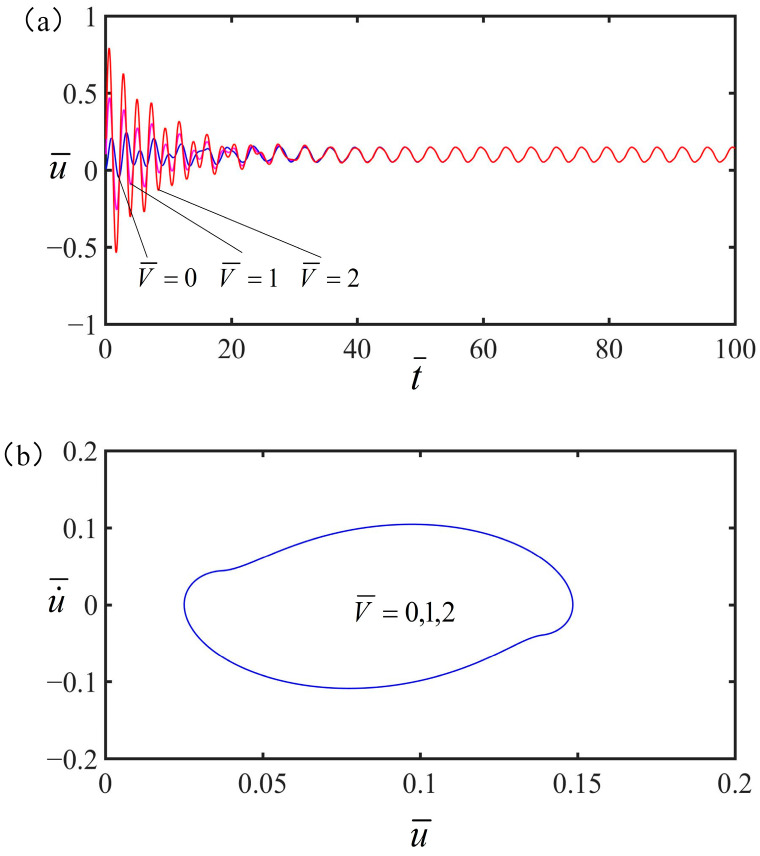
The effect of initial velocity on the vibration of the spring vibrator. In this case, other parameters are $\bar{q}=0.5$, $\bar{C}=0.25$, α=0.2, $\bar{g}=1.2$, $\bar{K}=8$, and ${\bar{S}}_{1}/\bar{S}=0.5$. (**a**) As the initial velocity increases, the amplitude of the forced vibration increases and eventually becomes stable. (**b**) Limit cycle.

Figure 12 presents the influence of the initial velocity on the vibration of the spring oscillator at the optimal electrothermal drive time rate. In the case, the parameters are $\bar{q}=0.5$, $\bar{C}=0.25$, α=0.2, $\bar{g}=1.2$, $\bar{K}=8$, and ${\bar{S}}_{1}/\bar{S}=0.5$. It is clear in Figure 12a that the vibration amplitude of the spring oscillator increases with the increase of initial velocity. Figure 12b indicates that when the initial velocity exists, the spring oscillator will be in a state of forced vibration, and the limit cycle remains unchanged. It can be seen that under the periodic electrothermal drive, the initial velocity only affects the initial amplitude, and then the vibration is stable.

### 4.7. An application Example of the Periodic Vibration of the Spring Oscillator

**Figure 13:**
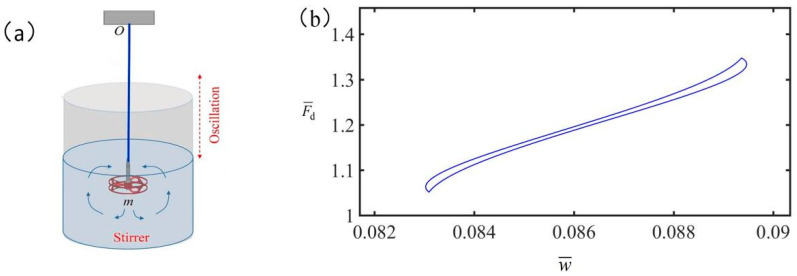
(**a**) The model of LCE agitator in solution. (**b**) The resistance of solution versus the displacement of agitator.

The spring oscillator system proposed in this paper can convert electrical energy into heat energy, which in turn is converted into mechanical energy. As shown in Figure 13a, the LCE spring oscillator can be placed in a transparent container of water connected to the stirrer. When the LCE spring oscillator is energized, the temperature rises to drive the rotation of the stirrer, and the electrical energy is converted into mechanical energy. In practical applications, the energy/power density and energy conversion efficiency are highly dependent on the specific energy conversion process. For the simple model established in this study, the damping energy is compensated by the electrical energy absorbed by the system during the periodic vibration of the LCE spring oscillator, and the work is carried out on the connected agitator. For such electrothermally driven devices, the work done by the system on the connected device can be considered as the effective work of the device.

Take the typical values as: damping coefficient C=8.02S⋅MPa/m, parallel current I=0.5A, and the wire resistance R=5Ω. The dimensionless parameters are set as α=0.2, $\bar{g}=1.2$, ${\bar{S}}_{1}/\bar{S}=0.5$, $\bar{K}=8$. The resistance of solution during agitation can be obtained by the formula $F_{d}=CV$, where V represents the speed of the mass, and C represents the damping coefficient. Figure 13b shows the relation between solution resistance and agitator displacement. The effective work done by the connected agitator in a period is $W=\int F_{d}d\bar{w}=1.14J$, where $\bar{w}$ represents the displacement of the mass, and the heat generated by electricity in a period can be calculated by the formula $Q=I^{2}R\Delta t=2.79J$, where Δt represents the electrothermal driving time in a period and Δt=2.23s. Then the efficiency of the work done by the system is $\eta =\frac{W}{Q}\times 100\%=40.14\%$.

## 5. Conclusions

In this paper, the forced vibration of LCE spring oscillator under periodic electrothermal drive is analyzed theoretically. Based on the LCE dynamic model, the dynamic model of LCE spring oscillator is established. The time history curves of the oscillator vibration is calculated numerically. Numerical results show that when the steady-state forced vibration of the spring oscillator is reached, the vibration period is the same as the power period. An optimal set of electrothermal drive time rate exists to maximize the vibration amplitude. The variations in the electrothermal drive time rate do not affect the optimal electrothermal drive period, the vibration period is equal to the electrothermal drive period. 

During forced vibration, the system amplitude is influenced by the adjustment of the electrothermal drive heat, damping coefficient, spring constant, gravital acceleration, shrinkage coefficient, etc., while the vibration amplitude gradually increases and becomes stable with the gradual increase of initial velocity. For example, the vibration amplitude decreases as the damping coefficient and spring constant increase, while it increases with the increasing gravital acceleration, shrinkage coefficient and electrothermal drive heat. The electrothermally driven vibration of LCE spring oscillator proposed in current paper has great potential for applications in soft robots, LCE-based electric locomotives and other fields.

## Figures and Tables

**Table 1:** Material properties and geometric parameters.

Parameter	Definition	Value	Unit
q	Electricity heat	$0\sim 7\times 10^{-3}$	J/s
c	Damping coefficient	$5\sim 20\times 10^{-3}$	kg/s
g	Gravital acceleration	10	${m/s}^{2}$
K	Spring constant	300~400	N/m
α	Shrinkage coefficient	0.2	/
v	Initial velocity	0~2.5	m/s
$L_{0}$	Original length of LCE fiber	0.08	m
*T*_e_	Ambient temperature	300	K
m	Mass of the ball	0.5	g
τ	Thermal relaxation time	0.1	s
k	Heat transfer coefficient	1	${W/m}^{2}\cdot K$
$\rho_{c}$	Specific heat capacity	0.1	J/kg⋅K

**Table 2:** Dimensionless parameters.

Parameter	$\bar{q}$	$\bar{C}$	$\bar{g}$	$\bar{K}$	α	$\bar{v}$
Value	0~0.7	1~4	1.2	6~8	0.2	0~2

## Data Availability

Not applicable.
